# Adjuvant systemic therapy in early breast cancer and results of a prospective observational multicenter BRIDE study: patients outcome and adherence to guidelines in cancer clinical practice

**DOI:** 10.3389/fonc.2025.1501667

**Published:** 2025-04-08

**Authors:** Stefania Gori, Alessandra Fabi, Catia Angiolini, Monica Turazza, Piermario Salvini, Gianluigi Ferretti, Elisabetta Cretella, Lorenzo Gianni, Claudia Bighin, Angela Toss, Claudio Zamagni, Patrizia Vici, Costanza De Rossi, Antonio Russo, Giancarlo Bisagni, Alessio Schirone, Lucia Borgato, Anna Cariello, Claudia Cappelletti, Roberto Bordonaro, Saverio Cinieri, Alessandra Modena, Matteo Valerio, Maria Francesca Alvisi, Irene De Simone, Francesca Galli, Eliana Rulli, Anna Santoni, Matteo Verzè, Fabrizio Nicolis

**Affiliations:** ^1^ Medical Oncology, IRCCS Sacro Cuore Don Calabria Hospital, Negrar di Valpolicella, Italy; ^2^ Precision Medicine Unit in Senology, Fondazione Policlinico Universitario A. Gemelli IRCCS, Rome, Italy; ^3^ Breast Unit and Multidisciplinary Oncology Group, Department of Breast Oncology, AOU Careggi, Florence, Italy; ^4^ Oncology, Humanitas Gavazzeni, Bergamo, Italy; ^5^ Oncological Medicine-Policlinico Ponte S Pietro di Istituti Ospedalieri Bergamaschi, Ponte San Pietro, Italy; ^6^ Division of Medical Oncology 1, IRCCS Regina Elena National Cancer Institute, Rome, Italy; ^7^ Medical Oncology-Azienda Sanitaria dell’Alto Adige, Bolzano, Italy; ^8^ Oncology-AUSL Romagna Rimini, Rimini, Italy; ^9^ Medical Oncology-IRCCS AOU San Martino IST, Genova, Italy; ^10^ Department of Oncology and Hematology, Azienda Ospedaliero-Universitaria di Modena, Modena, Italy; ^11^ Department of Medical and Surgical Sciences, University of Modena and Reggio Emilia, Modena, Italy; ^12^ Medical Oncology of Senology and Gynecology, IRCCS AOU Bologna, Policlinico Sant’Orsola, Bologna, Italy; ^13^ UOSD Sperimentazioni Fase IV, IRCCS Istituto Nazionale Tumori Regina Elena, Rome, Italy; ^14^ Medical Oncology-Ospedale dell’Angelo Azienda ULSS 3 Serenissima, Venice, Italy; ^15^ Medical Oncology, AOU Policlinico P. Giaccone, Palermo, Italy; ^16^ Medical Oncology, Comprehensive Cancer Centre, AUSL-IRCCS di Reggio Emilia, Reggio Emilia, Italy; ^17^ Clinical Oncology, Sant’Anna University Hospital, Ferrara, Italy; ^18^ Department of Oncology, San Bortolo General Hospital, Azienda ULSS8 Berica, Vicenza, Italy; ^19^ Medical Oncology, AUSL Romagna, Ravenna, Italy; ^20^ Medical Oncology, Fano Hospital, Fano, Italy; ^21^ Medical Oncology, ARNAS Garibaldi Hospital, Catania, Italy; ^22^ Medical Oncology, Antonio Perrino Hospital, Brindisi, Italy; ^23^ Laboratory of Methodology for Clinical Research, Department of Clinical Oncology, Istituto di Ricerche Farmacologiche Mario Negri IRCCS, Milan, Italy; ^24^ Medical Direction, IRCCS Sacro Cuore Don Calabria Hospital, Negrar di Valpolicella, Italy

**Keywords:** adjuvant systemic therapy, early breast cancer, outcome, adherence to guideline, quality indicators

## Abstract

**Introduction:**

Evaluation of every breast cancer (BC) patient by multidisciplinary team and application of guidelines are very important to ensure the best treatment and achieve the best outcome.

**Methods:**

The multicenter prospective observational BRIDE study enrolled, from 01/2018 to 02/2021, 1633 BC patients from 19 Italian cancer centers. To evaluate the clinical and biopathological characteristics of BC patients with pathological stage I-II-III treated with surgery followed by adjuvant systemic therapy, type of therapies delivered, outcome and adherence to guidelines, an analysis of 1123 patients out of 1633 patients enrolled in BRIDE study was conducted.

**Results:**

The 1123 patients with stage I-II-III BC had a median age of 61.2 years (Q1-Q3: 50.6-71.7); 70.2% were postmenopausal, 92.1% had ECOG PS 0, 68.4% pT1 disease, 70.7% pN0, 91.7% pathological stage I-II; 68.9% underwent conservative breast surgery and 79.8% sentinel lymph node biopsy alone. According to phenotypic subgroup, 80.6% of patients had a HER2-negative/HR-positive, 10.4% HER2-positive/HR-positive, 6.4% triple negative and 2.6% HER2-positive/HR-negative BC. In clinical practice, the phenotypic tumoral subgroup influenced oncologists in the choice of the type of adjuvant systemic therapy (p<0.0001) according to ESMO and AIOM Guidelines. Adjuvant radiotherapy was administered to 85.5% patients undergoing breast-conserving surgery. At the median follow up of 41.4 months (Q1: 35.3 months – Q3: 57.9 months), the DFS at 48 months was 92.8%, with different rates in the phenotypic subgroups. The adherence to AIOM Guidelines in clinical practice was ≥ 70% for the four evaluated quality indicators of treatment process.

**Discussion:**

In patients with pathological stage I-II-III BC, the phenotypic subgroup influenced the oncologists’ decision on the choice of type of adjuvant systemic therapy, as also indicated by international and national guidelines. In our patients, the DFS rate at 24 and 48 months after surgery was 95.4% and 92.8% respectively. The adherence to the AIOM Guidelines in clinical practice was high but having both quality indicators (shared at international and national level) to evaluate the quality of care in BC and standardized threshold levels to evaluate adherence to guidelines is very important today because this type of evaluation will increase in the coming years.

## 1 Introduction

Breast cancer (BC) is the most common cancer among women in Europe (1) and Italy (2), with 355,457 new cases in Europe and 54,976 in Italy in 2020. It also the first cause of cancer mortality among women, with 91,826 deaths in Europe and 12,995 in Italy (1, 3).

Due to the high incidence and good prognosis [5-year survival rate is 81.8% in Europe (4) and 87% in Italy (2)], its prevalence is high, with approximately 12.2 million survivors in Europe and 834,154 in Italy in 2020 (2).

The integration of the various therapeutic modalities (surgery, radiotherapy, systemic treatment, supportive therapies) and the collaboration of different specialists within multidisciplinary teams are fundamental to ensure the best treatment for each patient and achieve the best outcome (5).

In pathological stage I-II-III breast cancer patients (not treated with neo-adjuvant therapy), the knowledge of the clinical and biological prognostic factors is very important in order to identify the most appropriate adjuvant treatment, in line with the of recommendations from guidelines of scientific societies such as ESMO (6) and AIOM (7, 8).

To assess the characteristics of early breast cancer patients treated in Italy with surgery followed by adjuvant therapy, to describe the types of therapies administered and outcome, an analysis was performed in pathological stage I-II-III patients enrolled in the BRIDE study. The degree of implementation of the AIOM breast cancer guidelines in clinical practice was also evaluated.

## 2 Materials and methods

The BRIDE study was an observational, prospective, multicenter study which evaluated the distribution of patients with a stage I-II-III breast cancer candidate to systemic neo-adjuvant therapy or upfront surgery followed by adjuvant therapy, the parameters which determined the choice of systemic neo-adjuvant therapy or surgery followed by adjuvant therapy and the type of (neo)-adjuvant systemic therapy; the type of first line therapy in stage IV patients, outcome and adherence to AIOM guideline v.2017.

Previously, we reported on the results in the neo-adjuvant setting (9).

In this analysis, we evaluated the type of adjuvant systemic therapies administered after surgery in patients with pathological stage I-II-III breast cancer who were not candidates for neoadjuvant therapy, and their survival. The degree of implementation of the AIOM breast cancer guidelines v.2017 (7) in clinical practice was also reported.

Inclusion criteria were the following: female sex; 18 years old or older at time of diagnosis; histological diagnosis of *in situ* (DCIS, LCIS) or invasive breast carcinoma; stage 0-I-II-III-IV patient (according to TNM v. VII) (10); availability of clinical and/or pathological parameters: Tumor (T), Node (N), Metastasis (M); availability of biological parameters: Grading, ER and PgR status, Ki67 value, HER2 status on primary tumor and/or metastatic lesion. According to the International Conference on Harmonisation/Good Clinical Practice [ICH/GCP], patients had to have signed the written informed consent before enrolment. No exclusion criteria were set for the BRIDE study.

Breast cancer was considered ER negative if <1% or 0% of tumor cell nuclei were immunoreactive. A similar principle was applied to PgR testing (11, 12).

HER2 status was considered negative if equal to 0 or 1+ by immunohistochemistry (IHC) or if 2+ by IHC and not amplified (FISH/SISH/CISH). HER2 status was considered positive if 3+ by IHC or 2+ by ICH and amplified (FISH/SISH/CISH) or if amplified (FISH/SISH/CISH) (13).

The patients were classified in four phenotypic subgroups based on HER2, ER and PgR values:

a. HER2-positive/HR-positive (HER2+/HR+) subgroup included the patients with HER2-positive and ER and/or PgR positive breast cancer cells;
b. HER2-positive/HR-negative (HER2+/HR-negative) subgroup included the patients with HER2-positive and ER and PgR negative breast cancer cells;
c. HER2-negative/HR-positive (HER2-negative/HR+) subgroup included the patients with HER2-negative and ER and/or PgR positive breast cancer cells;
d. Triple negative (TN) subgroup included the patients with HER2-negative and ER and PgR negative breast cancer cells.

Primary endpoints of the BRIDE study were: percentage of patients with a stage I-II-III breast cancer eligible to initiate a neoadjuvant therapy and upfront surgery followed by systemic adjuvant therapy; proportion of patients who started neoadjuvant therapy; evaluation of clinical and biopathological characteristics that influenced the physician choice between neoadjuvant therapy or upfront surgery followed by adjuvant systemic therapy; frequencies with which different (neo)-adjuvant systemic regimens were chosen; frequencies of the different types of first-line treatment administered in the stage IV patients.

Secondary endpoints included disease-free survival; progression-free survival; overall survival; proportion of patients treated according to the AIOM breast guidelines v. 2017.

The protocol was reviewed by the independent ethic committee of the coordinating center and by the ethic committees of each participating center (14). The protocol complied with the recommendations of the 18th World Health Congress (Helsinki, 1964) (15).

This analysis included clinical and biopathological characteristics of stage I-II-III breast cancer patients treated with adjuvant systemic therapy after surgery; types of adjuvant systemic therapies chosen; disease-free survival (defined as the time from surgery to the first among the following events: local or regional relapse, distant metastasis, contralateral breast cancer, other invasive cancer different than breast, death) and overall survival (defined as the time from surgery to the time of death from any cause); the adherence to AIOM Breast Cancer Guidelines v.2017 in clinical practice (9).

To evaluate the adherence to AIOM breast guidelines v. 2017, the following indicators were assessed:

- percentage of patients with invasive stage I-II cancer and negative axillaries lymph nodes (not neoadjuvant candidate) subjected to a sentinel node biopsy;
- percentage of patients with invasive stage I-II cancer subjected to adjuvant therapy after conservative surgery;
- percentage of patients (no prior neoadjuvant therapy) with an interval between surgery and the beginning of the adjuvant systemic therapy ≤ 8 weeks;
- percentage of patients with positive hormone-receptors treated with adjuvant hormone-therapy.

### 2.1 Sample size determination

No formal statistical hypothesis for comparison was planned. It was estimated that 150 to 300 patients per center, per year would be available. According to the guidelines’ compliance objective, an agreement not lower than 80% approximately was expected. Assuming 50% to 100% variability in prevalence in each subgroup of patient populations (stage 0-I-II-III, stage IV), the precision of the statistical estimates (defined by the width of confidence interval of 95%) was calculated to vary between 3% and 5%.

According to these considerations at least 4500 patients’ data had to be obtained. Because of a low accrual rate observed mainly due to the Covid-19 pandemic, enrollment was stopped prematurely and the planned sample size was not reached.

### 2.2 Data collection and evaluated variables

The source of data was patients’ medical records. Patients’ demographic and clinical information, tumor characteristics, biological characterization and information regarding the treatments (adjuvant, neoadjuvant and metastatic settings) were collected as pseudonymized data and analyzed.

### 2.3 Statistical analysis

Tumor and patient’s characteristics were described through descriptive analysis. Continuous variables were described by median, first and third quartiles and minimum and maximum values (range). Categorical variables were described using the frequency and percentage of patients in each category.

The associations between the type of adjuvant therapy and the phenotypic subgroup were assessed by means of Chi-squared tests or Fisher test when appropriate. P-values <0.05 were considered statistically significant. The analysis was carried out using the SAS (Statistical Analysis System, SAS Institute, Version 9.4, Cary, NC) software.

## 3 Results

**Figure 1:**
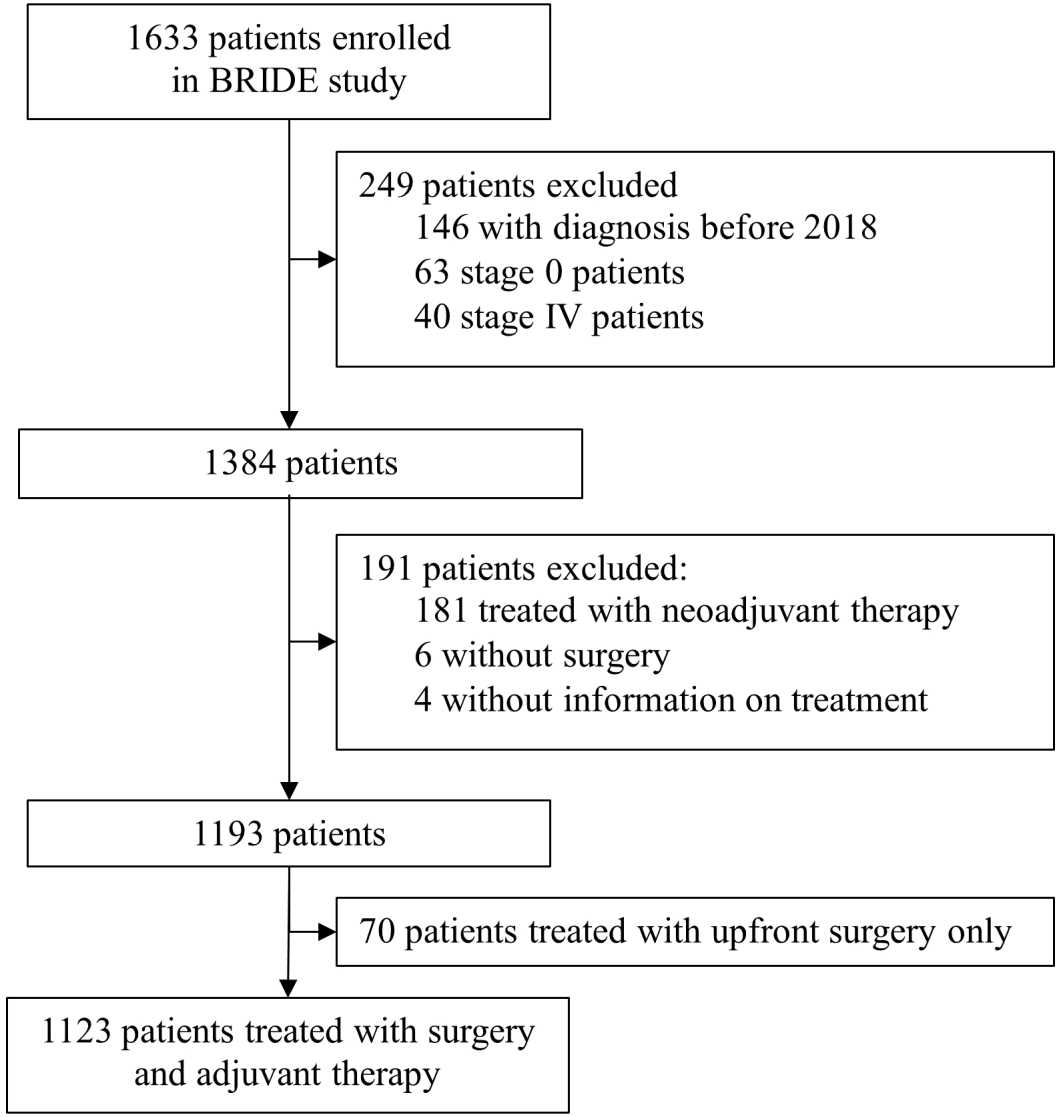
Study profile.

From January 8th, 2018 to February 3rd, 2021, 1633 patients with diagnosed breast cancer were enrolled in the BRIDE study from 19 Italian cancer centers. This analysis evaluated 1123 patients at pathological stage I-II-III treated with surgery and adjuvant therapy whose information on pathological stage at diagnosis (**Figure 1**) was available. The data snapshot for this analysis was carried out on June 3, 2024.

The median follow up is 41.4 months (First quartile: 35.3 months - Third quartile: 57.9 months)

All the 1123 pathological stage I-II-III patients were treated with upfront surgery followed by adjuvant systemic therapy. The clinical and tumor characteristic were summarized in **Table 1**.

**Table 1:** Patients with stage I-II-III invasive breast cancer treated with surgery and adjuvant systemic therapy: clinical and biopathological characteristics.

CHARACTERISTIC	Overall (N = 1123)
Age (years)	
Mean (SD)	61.3 (12.8)
Median (Q1 - Q3)	61.2 (50.6-71.7)
Min - Max	24.9 - 94.4
*Missing*	*2*
**Age ≥ 75 years old – n (%)**	190 (16.9%)
Menopausal status – n (%)	
Postmenopausal	785 (70.2%)
Premenopausal	333 (29.8%)
*Missing*	*5*
Performance status (ECOG) – n (%)	
0	1008 (92.1%)
1	62 (5.7%)
≥ 2	24 (2.2%)
*Missing*	*29*
Pathological tumor size – n (%)	
pT1	768 (68.6%)
pT2	314 (28.0%)
pT3	37 (3.3%)
pT4	1 (0.1%)
*Missing*	*3*
Pathological nodal status – n (%)	
pN0	788 (70.7%)
pN1 micrometastasis	60 (5.4%)
pN1	185 (16.6%)
pN2	52 (4.7%)
pN3	30 (2.7%)
*Missing*	*8*
Pathological stage – n (%)	
I	636 (57.1%)
II	386 (34.6%)
III A	61 (5.5%)
III B/C	31 (2.8%)
*Missing*	*9*
Histotype – n (%)	
Ductal	876 (78.0%)
Lobular	167 (14.9%)
Other*	80 (7.1%)
Grading – n (%)	
G1	234 (21.2%)
G2	580 (52.6%)
G3	289 (26.2%)
*Missing*	*20*
Ki-67 value – n (%)	
< 20%	591 (57.4%)
≥ 20%	438 (42.6%)
*Missing*	*94*
Phenotypic subgroup – n (%)	
HER2 negative/HR positive	898 (80.6)
HER2 positive/HR positive	116 (10.4)
Triple negative	71 (6.4%)
HER2 positive/HR negative	29 (2.6%)
*Missing*	*9*

N, number of subjects; Q1-Q3, First - third quartile; Min-Max, minimum - maximum values.

*Other = Mucinous, ductal + lobular, cribriform histotype; Hormonal receptor status cut off: 1%.

HER2 status positive if: IHC 3+ or IHC 2+ and amplified (by FISH/SISH/CISH) - amplified (by FISH/SISH/CISH).

Patients treated with surgery and adjuvant therapy had a mean age of 61.3 years, ECOG PS equal to 0 in 92.1% of cases. Tumors of 2 cm or less (pT1) were present in 68.4% of patients and a negative lymph node pathological status (pN0) was reported in 70.7% of cases. Pathological stage I-II was reported in most patients (91.7%).

According to phenotypic subgroup, 80.6% of patients (898) had a HER2-negative/HR-positive, 10.4% (116) HER2-positive/HR-positive, 6.4% (71) triple negative and 2.6% (29) HER2-positive/HR-negative breast cancer.

The proportion of patients with HER2-positive disease was 13.0%.

### 3.1 Surgery

Among the 1123 patients, 774 (68.9%) underwent conservative breast surgery and 349 (31.1%) underwent mastectomy.

Overall, axillary surgery was performed in 1052 (93.6%) of 1123 patients (**Table 2**).

**Table 2:** Surgery in patients with stage I-II-III invasive breast cancer.

CHARACTERISTIC	Adjuvant therapy (N = 1123)
BREAST SURGERY	
**Patients treated with breast surgery – n (%)**	1123 (100.0%)
Patients underwent - n (%)	
Conservative surgery	774 (68.9%)
Mastectomy	349 (31.1%)
AXILLARY SURGERY	
**Patients treated with axillary surgery – n (%)**	1052 (93.6%)
Type of axillary surgery – n (%)	
Only sentinel lymph node biopsy (SLNB)	830 (78.9%)
Only axillary lymph node dissection (ALND)	62 (5.9%)
ANLD after SLNB	150 (14.2%)
Unknown	10 (0.9%)
*Missing*	*71*

N, number of subjects.

Sentinel lymph node biopsy (SLNB) only was performed in 830 of 1052 patients (78.9%).

Axillary lymph node dissection alone was done in 62 (5.9%) of 1052 patients. Sixty patients (98.4%) out of 61 with information on dissection results, had positive axillary lymph-node.

Axillary lymph node dissection after SLNB was done in 150 (14.2%) out of 1052 patients (**Table 2**). Of these, 110 patients (75.3%) had other positive lymph-nodes at dissection.

Axillary surgery was not performed in 71 out of 1052 patients (6.4%) for unknown reasons: 29 out of these 71 patients (40.8%) were 70 years or older.

### 3.2 Type of adjuvant systemic therapy

The type of adjuvant systemic therapy administered differed according to the phenotypic tumor subgroup. As shown in **Table 3**, among the 1114 patients treated with adjuvant therapy, the type of systemic therapy administered was associated with the phenotypic subgroup (p <0.0001). Chemotherapy with anti-HER2 agent was administered to 79 (70.5%) HER2+/HR+ patients and to 25 (100.0%) HER2+/HR-negative breast cancer patients. Five patients with HER2+ disease not received anti-HER2 agent for unknown reasons. Chemotherapy alone was delivered to 177 (20.4%) HER2-negative/HR+ patients and to all 63 (100%) patients with a triple negative breast cancer.

**Table 3:** Type of adjuvant systemic therapy started after surgery, according to breast cancer phenotypic subgroup in 1114* patients.

CHARACTERISTIC	HER2+/HR+	HER2+/HR-	HER2-/HR+	Triple negative	Overall
	N = 116	N = 29	N = 898	N = 71	N = 1114*
Adjuvant systemic therapy – n (%)					
CT	5 (4.5%)	0 (0.0%)	177 (20.4%)	63 (100.0%)	245 (23.0%)
CT + AntiHER2 agent(s)	79 (70.5%)	25 (100.0%)	0 (0.0%)	0 (0.0%)	104 (9.7%)
Adjuvant ET administered – n (%)					
No	14 (12.3%)	25 (100%)	13 (1.5%)	63 (100%)	115 (10.8%)
Yes	100 (87.7%)	0 (0.0%)	852 (98.5%)	0 (0.0%)	952 (89.2%)
ET after surgey (ET alone)	28/100 (28.0%)	0 (0.0%)	690/852 (81.0%)	0 (0.0%)	718/952 (75.5%)
ET after CT (± antiHER2)	72/100 (72.0%)	0 (0.0%)	162/852 (19.0%)	0 (0.0%)	234/952 (24.5%)
*Missing*	*4*	*4*	*31*	*8*	*47*

*For 9 patients, out of 1123 treated with surgery and adjuvant therapy, the information on breast cancer phenotypic subgroup was missing.

N, number of subjects; CT, chemotherapy; ET, endocrine therapy. Hormonal receptor status cut off: 1%. HER2 status positive if: IHC 3+ or IHC 2+ and amplified (by FISH/SISH/CISH) or amplified (by FISH/SISH/CISH).

Endocrine therapy alone was administered after surgery in 28% of HER2+/HR+ patients and in 81.0% of HER2-negative/HR+ patients (**Table 3**). **Table 3** shows also the percentages of patients treated with endocrine therapy alone (started after surgery) or with endocrine therapy administered after chemotherapy (with or without anti-HER2 agent), according to breast cancer phenotypic subgroup.

In 14 patients (12.3%) with HR+/HER2+ breast cancer was not administered endocrine therapy: in 3 patients due to values of ER and PgR less than or equal to 10% and in other 11 patients due to unexplained causes. However, 7 of these 11 patients had pT1pN0 breast cancer and had received adjuvant chemotherapy with an anti-HER2 agent.

**Table 4** reports the adjuvant chemotherapy regimens administered (with or without anti-HER2 agent) according to breast cancer phenotypic subgroup. Out of 349 patients treated with chemotherapy regimens, anthracycline and taxane-based regimen was the most frequently used chemotherapy regimen in all subgroups (both in pN0 and pN-positive): for 43/84 (51.2%) HER2+/HR+ patients, 11/25 (44.0%) HER2+/HR-negative patients, 143/177 (80.8%) HER2-negative/HR+ patients, and 47/63 (74.6%) triple negative breast cancer patients.

**Table 4:** Adjuvant chemotherapy regimens (with or without Anti-HER2 agent) administered according to breast cancer phenotypic subgroup in 349 patients.

CHARACTERISTIC	HER2+/HR+	HER2+/HR-	HER2-/HR+	Triple negative	Overall
Patients treated with CT with or without Anti-HER2 agent	N = 84	N = 25	N = 177	N = 63	N = 349
Type of adjuvant CT with or without Anti-HER2 agent - n (%)					
Anthra-based	2 (2.4%)	0 (0.0%)	8 (4.5%)	6 (9.5%)	16 (4.6%)
Taxane-based	0 (0.0%)	0 (0.0%)	17 (9.6%)	7 (11.1%)	24 (6.9%)
Anthra and taxane-based	3 (3.6%)	0 (0.0%)	143 (80.8%)	47 (74.6%)	193 (55.3%)
Anthra-based + AntiHER2 agent	0 (0.0%)	2 (8.0%)	0 (0.0%)	0 (0.0%)	2 (0.6%)
Taxane-based + AntiHER2 agent	38 (45.2%)	14 (56.0%)	0 (0.0%)	0 (0.0%)	52 (14.9%)
Anthra and taxane-based + AntiHER2 agent	40 (47.6%)	9 (36.0%)	0 (0.0%)	0 (0.0%)	49 (14.0%)
CT no anthra or taxane based	0 (0.0%)	0 (0.0%)	9 (5.1%)	3 (4.8%)	12 (3.4%)
CT no anthra-taxane based + AntiHER2 agent	1 (1.2%)	0 (0.0%)	0 (0.0%)	0 (0.0%)	1 (0.3%)

N, number of subjects; CT, Chemotherapy; RT, Radiotherapy. Hormonal receptor status cut off: 1%. HER2 status positive if: IHC 3+ or IHC 2+ and amplified (by FISH/SISH/CISH) or amplified (by FISH/SISH/CISH).

The median time between surgery and start of adjuvant therapy (chemotherapy± anti-HER2 agent or endocrine therapy) was 5.4 weeks (first quartile: 3.6, third quartile: 7.6). The adjuvant therapy (chemotherapy± anti-HER2 agent or endocrine therapy) was started within 8 weeks for 835/1123 (74.4%) patients.

The median time between surgery and start of adjuvant chemotherapy was 7 weeks (Q1- Q3:5.7-8.9); in 233/349 (66.7%) patients adjuvant chemotherapy was started within 8 weeks.

The median time between surgery and start of adjuvant hormonal therapy was 4.3 weeks (first quartile: 3 weeks, third quartile: 6.7 weeks); in 600 of 719 (83.4%) patients adjuvant hormonal therapy was started within 8 weeks.

Adjuvant radiotherapy was administered to 654 (85.5%) of the 765 patients undergoing breast-conserving surgery (missing data in 13 patients) and was not performed in 111 patients (14.5%). Of these 111 patients undergoing conservative surgery who did not receive adjuvant radiotherapy, 17 (15.3%) patients had advanced age (≥75 years) and 11 (9.9%) patients had an ECOG performance status higher or equal to 1.

Adjuvant radiotherapy was administered to 72 (20.9%) out of 345 (missing data in 4 patients) patients treated with mastectomy.

### 3.3 Implementation of AIOM guidelines v.2017

To evaluate the implementation of the AIOM breast guidelines v. 2017 (7), some indicators were assessed in 1123 patients with stage I-II-III breast cancer not candidate to neoadjuvant therapy and treated with surgery followed by adjuvant systemic therapy, enrolled in the BRIDE study.

1. Percentage of patients with invasive stage I-II cancer and negative axillary lymph nodes (not neoadjuvant candidate) subjected to a sentinel node biopsy.
  Out of 809 patients with invasive stage I-II breast cancer and negative axillary lymph nodes (not candidate to neo-adjuvant therapy), 719 (88.9%) underwent sentinel node biopsy.
2. Percentage of patients with invasive stage I-II cancer treated with adjuvant therapy (chemotherapy± anti-HER2 agent or endocrine therapy) after conservative surgery.
  Out of 929 patients with invasive stage I-II breast cancer who underwent conservative breast surgery, 663 (71.4%) were treated, after surgery, with adjuvant systemic therapy (chemotherapy± anti-HER2 agent or endocrine therapy).
3. Percentage of patients (no prior neoadjuvant therapy) with the interval between surgery and the beginning of the adjuvant systemic therapy ≤ 8 weeks.
  Out of 1123 who were treated with adjuvant therapy after surgery, 835 (74.4%) started adjuvant therapy (chemotherapy± anti-HER2 agent or endocrine therapy) within 8 weeks from surgery.
  Specifically, 67.0% of patients (234/349) started chemotherapy within 8 weeks and 83.4% (600/719) endocrine therapy within 8 weeks.
4. Percentage of patients with positive hormone-receptors treated with adjuvant endocrine-therapy.
  Endocrine adjuvant therapy (alone or after chemotherapy ± anti-HER2 agent) was administered to 952 of 979 (97.2%) patients with HR-positive disease.

Other quality indicators were also evaluated in this analysis, such as the percentage of patients (without previous neoadjuvant therapy) treated with adjuvant endocrine therapy alone, who received adjuvant radiotherapy ≤ 12 weeks after surgery. After surgery (breast-conserving surgery or mastectomy), 43.8% (315/7129) of the patients treated with endocrine therapy alone, started adjuvant radiotherapy within 12 weeks after surgery, and specifically 59.0% (309/524) after breast-conserving surgery. These percentages were higher if the time interval increased to 16 weeks: 54.4% (391/719) of patients started radiotherapy after surgery and 72.0% (377/524) after breast-conserving surgery.

### 3.4 Outcome

At a median follow up of 41.4 months (first quartile: 35.3 months - third quartile: 57.9 months), out of 1114 patients 49 (4.4%) were found to have recurrences of disease, 19 (1.7%) other invasive cancers, 13 (1.2%) contralateral breast cancers and 12 (1.1%) deaths.

The percentage of patients who experienced an event was higher in the HER2+/HR-negative (20.7%) and in triple negative (11.3%) subgroups in comparison with the HER2-positive/HR-positive subgroup (7.8%) and the HER2-negative/HR+ subgroup (6.1%) (**Table 5**).

**Table 5:** Disease-free event and DFS rates at 24 and 48 months according to breast cancer phenotypic subgroup.

CHARACTERISTIC	HER2+/HR+	HER2+/HR-	HER2-/HR+	Triple negative	Overall
All patients	N = 116	N = 29	N = 898	N = 71	N = 1114
Event of DFS – n (%)					
No	107 (92.2%)	23 (79.3%)	843 (93.9%)	63 (88.7%)	1036 (93.0%)
Yes	9 (7.8%)	6 (20.7%)	55 (6.1%)	8 (11.3%)	78 (7.0%)
Type of DFS event – n (%)	N = 9	N = 6	N = 55	N = 8	N = 78
Relapse or distant metastasis	4/9 (44.4%)	6/6 (100. 0%)	30/55 (54.5%)	6/8 (75.0%)	46/78 (59.0%)
Contralateral breast cancer	3/9 (33.3%)	0/6 (0.0%)	10/55 (18.2%)	0/8 (0.0%)	13/78 (16.7%)
Other invasive cancer	1/9 (11.1%)	0/6 (0.0%)	12/55 (21.8%)	2/8 (25.0%)	15/78 (19.2%)
Death	1/9 (11.1%)	0/6 (0.0%)	3/55 (5.5%)	0/8 (0.0%)	4/78 (5.1%)
**DFS rate at 24 months**	95.6%	84.2%	96.0%	91.1%	95.4%
**DFS rate at 48 months**	94.0%	84.2%	93.4%	86.3%	92.8%

N, number of subjects; DFS, disease-free survival was defined as the time from surgery to the first among the following events: local or regional relapse, distant metastasis, contralateral breast cancer, other invasive cancer different than breast, death.

**Figure 2:**
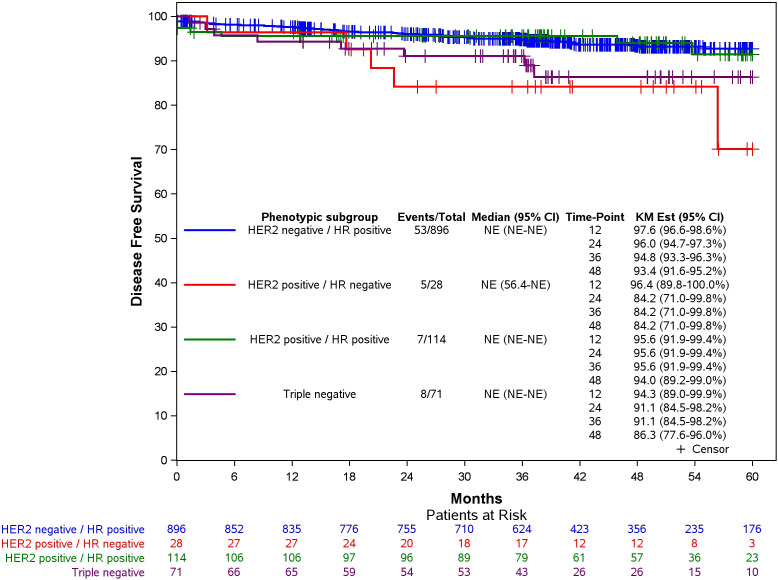
Disease-Free survival according to phenotypic subgroup in patients with stage I-II-III breast cancer (in 1109 patients with available recurrence date).

**Figure 2** shows the disease-free survival curves according to phenotypic subgroup for the 1109 patients with available recurrence date.

Moreover, the DFS rates at 24 and 48 months are provided for the different phenotypic subgroups of breast cancer in **Table 5**.

At a median follow up of 41.4 months (95% CI: 39.9-45.1) 12 deaths were reported; the overall survival rate at 24 and 48 months in the all patients was of 99.8% and 98.8%, respectively.

## 4 Discussion

This study reported the results of the analysis of 1123 patients with pathological stage I-II-III breast cancer enrolled in the BRIDE trial from January 2018 to February 2021 from 19 Italian cancer centers and treated with upfront surgery followed by adjuvant systemic therapy.

These 1123 patients had a median age of 61.2 years, small tumors (pT1) in 68.4% of cases and a negative lymph node pathological status (pN0) in 70.7%; pathological stage I-II was reported in 91.7% of patients. These characteristics reflect a good implementation of mammographic screening in women aged 50-69 years in Italy (16) and also the high degree of awareness of women with regard to breast cancer.

The percentage of patients with HER2-positive disease was 13.0%, which is in line with what has been reported in the literature (17).

Conservative breast surgery was performed in 68.9% of early breast cancer patients enrolled in the BRIDE trial from January 2018 to February 2021: this percentage overlaps with the Italian national average reported by the National Agency for Regional Health Services (Agenas) for the year 2018 (68.22%) and for the year 2019 (67.74%) (18).

In 1123 patients with pathological stage I-II-III breast cancer treated with adjuvant systemic therapy, the phenotypic subgroup influenced the oncologists’ decision on the choice of adjuvant systemic therapy type in a statistically significant (p < 0.0001) way, as recommended by ESMO (6) and AIOM guidelines (7, 8). The most frequently administered chemotherapy regimen was the anthracycline- and taxane-based regimen.

At the median follow up of 41.4 months (Q1: 35.3 months – Q3: 57.9 months), the DFS at 24 and 48 months after surgery was 95.4% and 92.8%, respectively, with different rates in the various phenotypic subgroups.

In this analysis, the grade of the concordance between AIOM breast cancer guidelines v.2017 (7) and clinical practice in Italy was also evaluated, because the implementation of guidelines increases patient survival (19). To assess adherence to breast cancer guidelines, we used some indicators from among those reported in previous Italian surveys (20, 21) and those proposed in Italy by the Ministry of Health in 2014 (22). We reported that 88.9% of patients with invasive stage I-II cancer and negative axillary lymph nodes (not candidate to neo-adjuvant therapy) underwent a sentinel node biopsy; 71.4% of patients with invasive stage I-II cancer were treated with adjuvant therapy after conservative surgery; 74.4% of patients started adjuvant therapy within ≤ 8 weeks; adjuvant endocrine therapy (alone or in combination with an anti-HER2 agent) was administered to 100% (952 out of 952) of patients with HR-positive disease. This percentage was higher than the 81% reported in Italy in 2003, when endocrine therapy was not prescribed in 19% of patients with HR-positive disease (23), and resulted also superior to that reported in previous assessments of guideline-adherence in Italian clinical practice carried out in 2007 (20) and 2011 (21).

The literature reports that the implementation of guideline recommendations within multidisciplinary teams is very important to optimize the quality of treatment and can lead to a reduction in breast cancer morbidity and mortality. The multidisciplinary team brings together the various specialists (oncologist, surgeon, radiation oncologist, radiologist, and pathologist, nurse) involved in the treatment of breast cancer patients, with the aim of identifying the most appropriate diagnostic and therapeutic pathways for the individual patients (5). The introduction of multidisciplinary teams in clinical practice has been associated with improved survival of breast cancer patients, with an 18% reduction in 5-year breast cancer mortality and an 11% reduction in all-cause mortality (24–26). Therefore, it is necessary that all breast cancer patients be discussed by multidisciplinary teams and that this activity is monitored regularly.

However, some data showed that that a substantial proportion of breast cancer patients did not receive the treatment recommended by the guidelines. In a systematic literature review that included 41 studies published between 1997 and 2019, conducted in eight European countries (including Italy), the median adherence to the guidelines for overall treatment processes (including surgery, chemotherapy, endocrine therapy and radiotherapy) was 57.7% (interquartile range -IQR- 38.8-67.3%), while for systemic therapy (chemotherapy and endocrine therapy) was 76% and for endocrine therapy 90% (IQR 87-92.5%) (27).

However, our study has some limitations.

Firstly, some information is missing. We do not know the reasons why axillary surgery was not performed in 71 patients (6.4%); data analysis revealed that 29 of these 71 (40.8%) were 70 years or older. There was also a lack of information as to why endocrinotherapy was not administered to 14 (12.3%) patients with HR+/HER2+ disease: re-evaluation of the database revealed that in 3 patients it had probably not been prescribed because the ER and PgR values were less than or equal to 10%. In addition, 5 (4.5%) patients with HER2+ breast cancer received chemotherapy but not an anti-HER2 agent, for unknown reasons.

Secondly, the indicators for assessing the adherence to the guidelines were chosen from those reported in previous Italian surveys and from those proposed in 2014 by the Italian Ministry of Health in the ‘Guidelines on the organizational and care modalities of the breast unit network’ approved by the State-Regions Conference on 18 December 2014, in response to a 2006 European directive committing all Member States to activate breast units in their national territory by 2016 (22). However, both in Europe and in Italy, the need for homogeneous indicators to assess adherence to the guidelines has been pointed out for many years, also for periodic evaluations of the activity carried out within the multidisciplinary teams. Indeed, the European Society of Breast Cancer Specialists (EUSOMA) in 2010 described a set of benchmark quality indicators (QIs) that could be adopted by breast centers for quality assurance purposes and to establish an agreed minimum standard of care (28). In 2017 (29) and 2024 (30) EUSOMA updated the paper on Quality Indicators and some of these QI are today utilized by National Health Service systems to evaluate the guideline adherence in breast cancer centers. In order to ensure quality of care for patients with breast cancer, the adherence to the guidelines, supported by the work of multidisciplinary and interdisciplinary teams, must be evaluated periodically in the context of healthcare service research, using shared quality indicators. Health professionals’ adherence to breast cancer guidelines in Europe must be improved in almost all treatment processes.

Guideline development and implementation processes should also address the main factors influencing health care providers’ adherence, particularly those related to the patient.

Thirdly, since the period analyzed in our study many aspects of clinical practice have changed significantly. The use of genomic testing or CDK4/6 inhibitors in luminal cancers, double anti-HER2 blockade in HER2-positive and neoadjuvant chemo-immunotherapy in triple-negative tumors are all factors that may make the indicators used somewhat outdated and probably do not reflect good clinical practice, as indicated in the recently published EUSOMA quality indicators update (30).

### 4.1 Conclusions

In patients with pathological stage I-II-III breast cancer, the phenotypic subgroup influenced the oncologists’ decision on the choice of type of adjuvant systemic therapy.

In these patients, the DFS rate at 24 and 48 months after surgery was very high, 95.4% and 92.8% respectively, but with different rates in the various phenotypic subgroups.

The adherence to the AIOM Guidelines v.2017 in clinical practice was ≥ 70% for the four evaluated quality indicators.

Despite the lack of nationally and internationally agreed quality indicators that can be used to assess the quality of care in early-stage breast cancer and the lack of standardized threshold levels to assess adherence to guidelines, the percentages reported in our observational, prospective, multicenter study demonstrated good adherence to the national guidelines.

The degree of adherence to the guidelines in the clinical practice must increase in the next years, and the quality indicators must be shared nationally and internationally so that the degree of quality provided to breast cancer patients can be uniformly assessed. The updating of quality indicators that EUSOMA has been carrying out since 2010 will be very helpful in this regard.

## Data Availability

The raw data supporting the conclusions of this article will be made available by the corresponding author upon reasonable request.
